# Phylogenetic relationships of '*Polyalthia*' in Fiji

**DOI:** 10.3897/phytokeys.165.57094

**Published:** 2020-10-28

**Authors:** Bine Xue, Yanwen Chen, Richard M.K. Saunders

**Affiliations:** 1 College of Horticulture and Landscape Architecture, Zhongkai University of Agriculture and Engineering, Guangzhou 510225, Guangdong, China; 2 Division of Ecology & Biodiversity, School of Biological Sciences, The University of Hong Kong, Pokfulam Road, Hong Kong, China

**Keywords:** Annonaceae, Fiji, *
Huberantha
*, molecular phylogeny, *
Polyalthia
*

## Abstract

The genus *Polyalthia* (Annonaceae) has undergone dramatic taxonomic changes in recent years. Nine *Polyalthia* species have historically been recognized in Fiji, all of which have subsequently been transferred to three different genera, viz. *Goniothalamus*, *Huberantha* and *Meiogyne*. The transfer of six of these species has received strong molecular phylogenetic support, although the other three species, *Polyalthia amoena*, *P. capillata* and *P. loriformis* [all transferred to *Huberantha*], have never previously been sampled in a phylogenetic study. We address this shortfall by sampling available herbarium specimens of all three species and integrating the data in a molecular phylogenetic analysis. The resultant phylogeny provides strong support for the transfer of these species to *Huberantha*. The taxonomic realignment of all nine Fijian species formerly classified in *Polyalthia* is also clearly demonstrated and supported by the resultant phylogeny. The updated taxonomic treatments of the nine species, a key to the three genera and a key to the Fijian *Huberantha* species are provided.

## Introduction

The genus *Polyalthia* Blume (Annonaceae) has historically been the source of considerable taxonomic confusion (van Setten and Koek-Noorman 1992; Doyle and Le Thomas 1994; Doyle et al. 2000). Recent molecular phylogenetic studies have confirmed its polyphyletic status, and accelerated the segregation of disparate elements from *Polyalthia* s.l., with distantly related species transferred to various genera, including *Fenerivia* Diels (Saunders et al. 2011), *Goniothalamus* (Blume) Hook.f. & Thomson (Tang et al. 2013), *Huberantha* Chaowasku (Chaowasku et al. 2012; Chaowasku et al. 2015), *Maasia* Mols, Keßler & Rogstad (Mols et al. 2008), *Marsypopetalum* Scheffer (Xue et al. 2011), *Meiogyne* Miquel (Xue et al. 2014), *Monoon* Miquel (Xue et al. 2012), *Polyalthiopsis* Chaowasku (Chaowasku et al., 2018; Xue et al., 2020a), *Wangia* X.Guo & R.M.K.Saunders (Xue et al. 2016), and *Wuodendron* B.Xue, Y.H.Tan & T.Chaowasku (Xue et al. 2018).

In Fiji, ten species were published under the name *Polyalthia* (Seemann 1861; Gillespie 1931; Smith 1936, 1950, 1978). In Smith’s (1981) revision of Fijian *Polyalthia*, nine species were accepted: *P. amoena* A.C.Sm., *P. amygdalina* (A.Gray) Gillespie, *P. angustifolia* A.C.Sm., *P. capillata* A.C.Sm., *P. habrotricha* A.C.Sm., *P. insularis* (A.C.Sm.) A.C.Sm., *P. laddiana* A.C.Sm., *P. loriformis* Gillespie, and *P. vitiensis* Seem. The tenth species, *P. pedicellata* A.C.Sm., was treated as a synonym of *P. vitiensis* (Smith 1981). Among the nine accepted species, *P. insularis* was later recognized as Meiogyne stenopetala (F.Muell.) Heusden subsp. insularis (A.C.Sm.) Heusden (van Heusden 1994), although this was recently elevated to species rank as *Meiogyne insularis* (A.C.Sm.)D.C.Thomas, B.Xue & R.M.K.Saunders (Xue et al. 2014). Three other Fijian *Polyalthia* species were subsequently transferred to *Meiogyne*: *P. amygdalina* as *Meiogyne amygdalina* (A.Gray) B.Xue & R.M.K.Saunders; *P. habrotricha* as *Meiogyne habrotricha* (A.C.Sm.) B.Xue & R.M.K.Saunders; and *P. laddiana* as *Meiogyne laddiana* (A.C.Sm.) B.Xue & R.M.K.Saunders. The above treatments were based on combined molecular and morphological evidence (Xue et al. 2014). A fifth species, *Polyalthia angustifolia*, was transferred to *Goniothalamus* as *G. angustifolius* (A.C.Sm.) B.Xue & R.M.K.Saunders (Tang et al. 2013); although the lack of flowers in the type specimen precluded an identification as *Goniothalamus* based on the connivent inner petals, the evidence from an unpublished molecular phylogeny based on sequences of the type specimen was strong enough to support the transfer (Xue 2013). The transfer was later supported in a published phylogenetic analysis with a larger taxon sampling of *Goniothalamus* (Tang et al. 2015). The remaining four species–*P. amoena*, *P. capillata*, *P. loriformis*, and *P. vitiensis*–were transferred to *Huberantha* based on a morphological study as *Huberantha amoena* (A.C.Sm.) Chaowasku, *H. capillata* (A.C.Sm.) Chaowasku, *H. loriformis* (Gillespie) Chaowasku, and *H. vitiensis* (Seem.) Chaowasku (Chaowasku et al. 2015) [initially under the generic name *Hubera* Chaowasku (Chaowasku et al. 2012), although this name was considered illegitimate (Chaowasku 2013; Applequist 2014)]. All nine Fijian *Polyalthia* species have therefore been realigned to three different genera.

Turner and Utteridge (2017) recently reviewed the taxonomy and distribution of Pacific Annonaceae and incorporated the above-mentioned treatments of Fijian *Polyalthia* species. It is noteworthy that although the treatment of five of the Fijian *Polyalthia* species has been supported by molecular studies (Xue 2013; Xue et al. 2014; Tang et al. 2015), the transfer of the other four species to *Huberantha* was only based on morphological comparison (Chaowasku et al. 2012, 2015). Thomas et al. (2015) sampled *Huberantha vitiensis* (as ‘*Hubera vitiensis*’) in their phylogenetic study while studying the origins of intercontinental disjunctions in Annonaceae, and confirmed its taxonomic affinity with *Huberantha*. The other three species have never previously been sampled in a phylogenetic study.

As the genus *Huberantha* is taxonomically challenging and difficult to recognize, the transfer for some species based on limited collections may be problematic in the absence of molecular evidence. One example is *Polyalthia floribunda* Jovet-Ast from Vietnam (Jovet-Ast 1940), which was transferred to *Huberantha* based on its cuneate, symmetrical leaf bases, single ovule per carpel and leaf venation pattern (Turner 2016). A recent molecular phylogenetic study has revealed that the species is not congeneric with *Huberantha*, however, but is sister to *Miliusa*, although without statistical support (Chaowasku et al. 2018). A new genus, *Polyalthiopsis* Chaowasku, was therefore erected to accommodate it (Chaowasku et al. 2018). The sister relationship between *Polyalthiopsis* and *Miliusa* was later supported by Xue et al. (2020a, b) and Chaowasku et al. (2020), redefining the long-recognized sister relationship between *Huberantha* and *Miliusa* in previous studies (Mols et al. 2008; Saunders et al. 2011; Xue et al. 2011, 2012; Chaowasku et al. 2012, 2014).

As nomenclatural transfers based solely on morphological data can sometimes be misleading, molecular phylogenetic data can provide invaluable evidence for confirming correct taxonomic placement. To avoid such errors, we have therefore sampled the remaining three Fijian *Huberantha* species and undertaken a phylogenetic study to confirm their taxonomic placements.

## Materials and methods

Three Fijian *Huberantha* species that lack DNA sequence data–*H. amoena*, *H. capillata* and *H. loriformis*–were sampled in this study to verify their generic position. The other six previously recognized Fijian ‘*Polyalthia*’ species were also included in this study. Sequence data for three commonly used chloroplast regions (*matK*, *rbcL* and *trnL-F*) were newly generated for the three *Huberantha* species. Sequences for other taxa were downloaded from the nucleotide database of the National Centre for Biotechnology Information (http://www.ncbi.nlm.nih.gov). The final data matrix comprised a total of 77 Annonaceae species, representing the major clades in the family. The samples, localities and GenBank accession numbers are listed in the Appendix 1.

The phylogenetic trees were reconstructed using Bayesian Inference (BI) and maximum likelihood (ML) methods. Detailed information regarding DNA extraction, PCR amplification, and primer sequences are available (Xue et al. 2011, 2012), as is information on sequence alignment, model selection of the sequence matrix constructed and methods in tree reconstruction (Xue et al. 2018).

## Results

**Figure 1.**
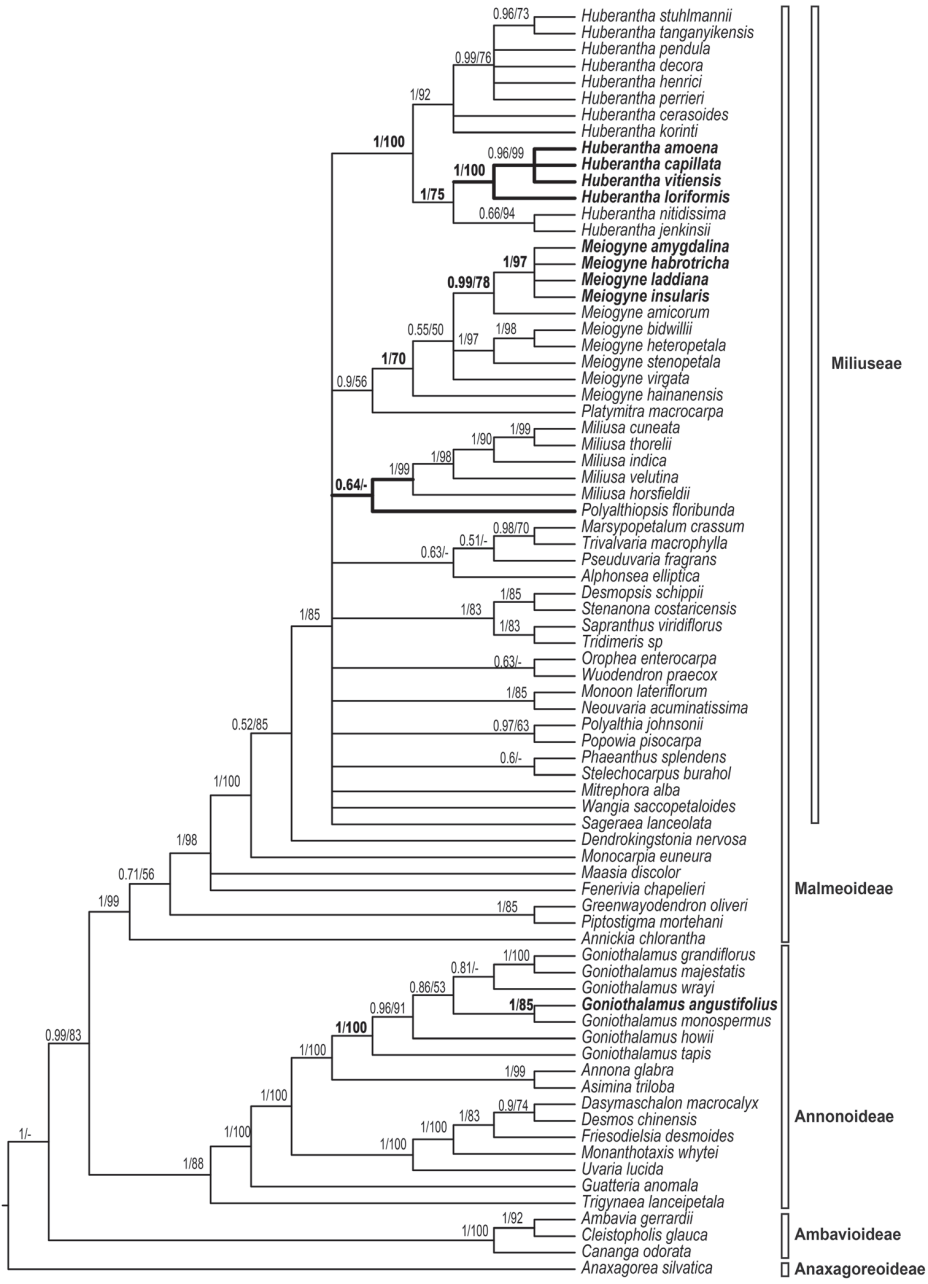
Bayesian 50% majority-rule consensus tree inferred from combined data of *matK*, *rbcL* and *trnL-F* under three-partitioned models. Numbers at the nodes indicate BI posterior probabilities and ML bootstrap values (> 50%). Species names of previous Fijian *Polyalthia* are in bold.

The concatenated alignment of the 77-taxon dataset consisted of 3,659 aligned positions (*trnL-F*: 1,475 bp; *matK*: 834 bp; and *rbcL*: 1,350 bp). The Bayesian and ML analyses resulted in similar topologies. The 50% majority-rule consensus tree resulting from the Bayesian analysis under the three-partitioned model is shown as Fig. 1. The results are consistent with previous phylogenetic analyses of the family in which the backbone of the tribe Miliuseae remains largely unresolved.

The Fijian species previously assigned to *Polyalthia* are retrieved in three distinct clades (Fig. 1). *Huberantha amoena*, *H. capillata*, *H. loriformis*, and *H. vitiensis* form a well-supported clade (PP = 1; ML BS = 100%) nested within *Huberantha* (PP =1; ML BS = 100%), with *H. nitidissima* (Dunal) Chaowasku and *H. jenkinsii* (Hook.f. & Thomson) Chaowasku forming the sister clade (PP =1; ML BS = 75%). *Polyalthiopsis* is recovered as sister to *Miliusa* instead of *Huberantha*, although lacking statistical support. *Meiogyne amygdalina*, *M. habrotricha*, *M. laddiana*, and *M. insularis* form a well-supported clade (PP = 1; ML BS = 97%) nested within *Meiogyne* (PP = 1; ML BS = 70%), with *Meiogyne amicorum* (A.C.Sm.) B.Xue & R.M.K.Saunders from Tonga being the closest sister clade (PP = 0.99; ML BS = 78%). *Goniothalamus angustifolius* is nested within the *Goniothalamus* clade (PP = 1; ML BS = 100%) and closely related to another *Goniothalamus* species in Fiji, *G. monospermus* (A.Gray) R.M.K.Saunders (PP = 1; ML BS = 85%).

## Discussion

The transfer of *Polyalthia amoena*, *P. capillata* and *P. loriformis* to *Huberantha* is supported here in a molecular phylogenetic analysis for the first time. The four Fijian *Huberantha* species form a well-supported clade that shows a close affinity with *H. nitidissima* (distributed in Papua New Guinea, Australia, and New Caledonia) and *H. jenkinsii* (distributed in continental Asia and western Malesia). The clade comprising *Polyalthia nitidissima* and the Fijian ‘*Polyalthia*’ species is well separated from its sister clade consisting of species from continental Asia, Africa and Madagascar; this is consistent with the phylogeny including only one Fijian *Huberantha* species (*H. vitiensis*) published by Thomas et al. (2015).

**Figure 2.**
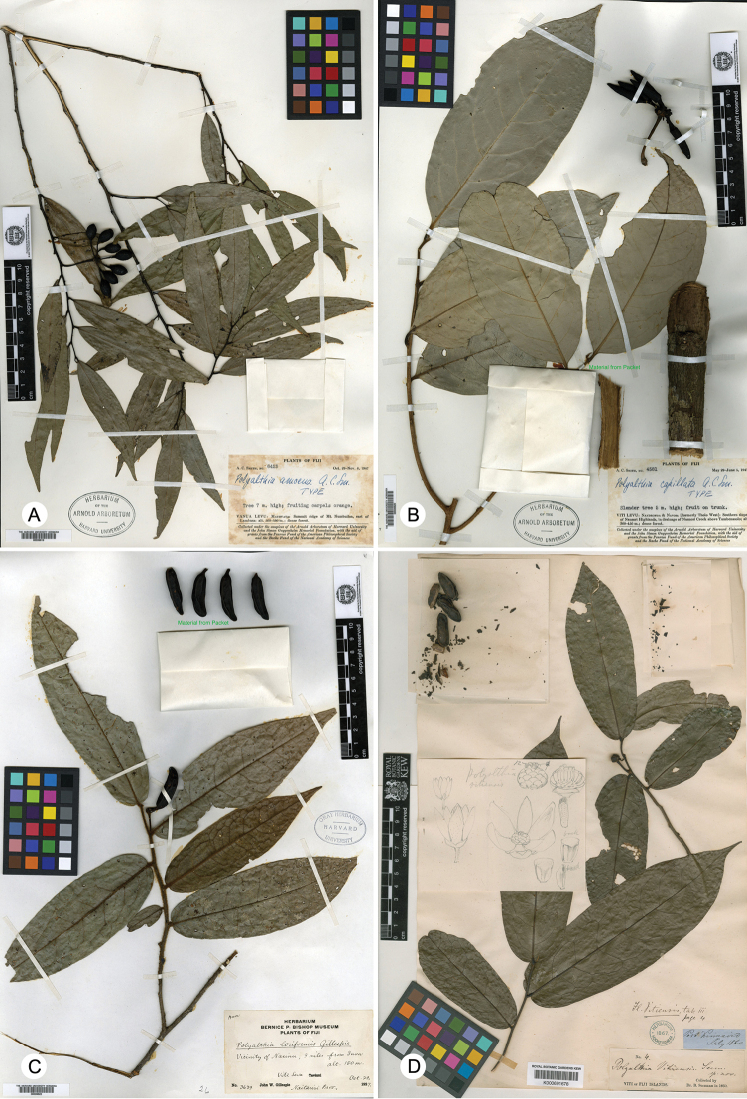
Type specimens of the four Fijian *Huberantha* species **A***Huberantha amoena* (*A.C. Smith 6423*, A) **B***H. capillata* (*A.C. Smith 4581*, A) **C***H. loriformis* (*J.W. Gillespie 3639*, GH) **D***H. vitiensis* (*B. Seemann 4*, K).

*Huberantha* can be distinguished from other closely related genera by a combination of characters, including leaves with reticulate tertiary venation, axillary inflorescences, a single ovule per ovary (and therefore single-seeded monocarps), seeds with a flat to slightly raised raphe, spiniform(-flattened peg) ruminations of the endosperm, and pollen with a finely and densely granular infratectum (Chaowasku et al. 2012). It resembles *Polyalthia* and *Polyalthiopsis* in having brochidodromous foliar venation with reticulate tertiary veins. *Polyalthia* differs in having a generally asymmetrical leaf base and ovaries with 2–6 ovules (Xue et al. 2012). *Polyalthiopsis* differs in having foliar glands, petioles with transverse striations when dry, a leaf midrib that is raised adaxially in vivo, ovaries with 1–2 ovules, and lamelliform endosperm rumination (Chaowasku et al. 2018; Xue et al. 2020a). These four Fijian *Huberantha* species all possess a symmetrical leaf base, a flat leaf midrib adaxially, axillary inflorescences and single-seeded monocarps (Fig. 2). It is noteworthy that the monocarps are much larger, however, especially in *P. capillata* (25–30 cm long, 0.5–1cm broad; Fig. 2B), *P. loriformis* (up to 4 cm long, 1 cm broad; Fig. 2C) and *P. vitiensis* (up to 4.5 cm long, 1.5 cm broad; Fig. 2D) (Gillespie 1931, Smith 1950). Other *Huberantha* species, including *H. nitidissima* and *H. jenkinsii*, have much smaller monocarps, with the largest dimension rarely exceeding 1 cm. Seed size is possibly correlated with various factors, including overall plant size and architecture, dispersal agents, habitat and insularity (Bellot et al. 2020): the larger fruits of these Fijian *Huberantha* species probably reflect adaptations to alternative dispersal vectors.

The taxonomic placement of *Goniothalamus angustifolius* and the four *Meiogyne* species (*M. amygdalina*, *M. habrotricha*, *M.insularis* and *M. laddiana*) are confirmed in our study (Fig. 1): *G. angustifolius* is sister to another Fijian *Goniothalamus* species, *G. monospermus*; and the four Fijian *Meiogyne* species form a well-supported clade, although the relationship among these species is not well resolved since our analysis is based on only three chloroplast regions. The topology is consistent with a better-resolved topology based on seven markers reconstructed by Thomas et al. (2012) and Xue et al. (2014), however, with the Fijian clade sister to *M. amicorum* from Tonga, together forming a well-supported clade within the Australian-Pacific clade of *Meiogyne* species (Thomas et al. 2012, Xue et al. 2014).

## Conclusions

The transfer of *Polyalthia amoena*, *P. capillata* and *P. loriformis* to *Huberantha* is supported here in a molecular phylogenetic study for the first time. The phylogenetic analyses of previous Fijian *Polyalthia* species confirm that this group is a highly heterogeneous assemblage, with nine species now divided into three distantly related genera, viz. *Goniothalamus*, *Huberantha* and *Meiogyne*. The updated taxonomic treatments of the nine species, a key to the three genera and a key to the four *Huberantha* species, are provided below.

### Taxonomic treatment of the nine previous Fijian *Polyalthia* species

#### Goniothalamus angustifolius

Taxon classificationPlantaeMagnolialesAnnonaceae

(A.C.Sm.) B.Xue & R.M.K.Saunders, PhytoKeys 32: 33. 2013.

6EB4450E-2874-582C-A313-B06B9472B40D

##### Basionym.

*Polyalthia angustifolia* A.C.Sm., Bull. Torrey Bot. Club 70: 538. 1943.

##### Type.

Fiji, Viti Levu, Naitasiri Province, Tamavua woods, 7 miles from Suva, 9 Aug. 1927, *J.W. Gillespie 2198* (holotype: A[A00039617]; isotypes: GH[GH00039618], BISH).

#### Huberantha amoena

Taxon classificationPlantaeMagnolialesAnnonaceae

(A.C.Sm.) Chaowasku, Kew Bulletin 70(2)–23: 2. 2015.

A781E929-5B81-557C-8D36-CFC6B982CA24

##### Basionym.

*Polyalthia amoena* A.C.Sm., Journal of the Arnold Arboretum 31: 159. 1950.

##### Homotypic synonym.

*Hubera amoena* (A.C.Sm.) Chaowasku, Phytotaxa 69: 47. 2012.

##### Type.

Fiji, Vanua Levu, Mathuata Province, east of Lambasa, on the summit ridge of Mt. Numbuiloa, 29 Oct. 1947, *A.C. Smith 6423* (holotype: A[A00039619]; isotypes: BISH, BRI[BRI-AQ0211645], K[K000691676], L[L0038107], P[P00636930], S[S-G-7470], US[US00098656]).

#### Huberantha capillata

Taxon classificationPlantaeMagnolialesAnnonaceae

(A.C.Sm.) Chaowasku, Kew Bulletin 70(2)–23: 2. 2015.

66589574-F06F-5DEA-B3C1-B2896F72D01F

##### Basionym.

*Polyalthia capillata* A.C.Sm., Journal of the Arnold Arboretum 31: 158. 1950.

##### Homotypic synonym.

*Hubera capillata* (A.C.Sm.) Chaowasku, Phytotaxa 69: 47. 2012.

##### Type.

Fiji, Viti Levu, Nandronga & Navosa Province, on the southern slopes of the Nausori Highlands, in the drainage of Namosi Creek, above Tumbenasolo, 29 May 1947, *A.C. Smith 4581* (holotype: A[A00039620]; isotypes: BISH, BRI[BRI-AQ0332771], K[K000691675], US[US00098658]).

#### Huberantha loriformis

Taxon classificationPlantaeMagnolialesAnnonaceae

(Gillespie) Chaowasku, Kew Bulletin 70(2)–23: 3. 2015.

23AD510C-B73F-52CF-8C05-DFB1FC8782F9

##### Basionym.

*Polyalthia loriformis* Gillespie, Bulletin of the Bernice P. Bishop Museum 83: 4, fig. 1. 1931.

##### Homotypic synonym.

*Hubera loriformis* (Gillespie) Chaowasku, Phytotaxa 69: 49. 2012.

##### Type.

Fiji, Viti Levu, Naitasiri Province, in the vicinity of Nasinu, 29 Oct. 1927, *J.W. Gillespie 3639* (holotype: BISH[BISH1011147]; isotypes: BISH[BISH1011148], GH[GH00039622], NY[NY00026209]).

#### Huberantha vitiensis

Taxon classificationPlantaeMagnolialesAnnonaceae

(Seem.) Chaowasku, Kew Bulletin 70(2)–23: 3. 2015.

3834FB12-DCAB-5BC9-9FED-AA5C195E0D09

##### Basionym.

*Polyalthia vitiensis* Seem., Flora Vitiensis 1: 4, pl. 3. 1865.

##### Homotypic synonym.

*Hubera vitiensis* (Seem.) Chaowasku, Phytotaxa 69: 51. 2012.

##### Heterotypic synonym.

*Polyalthia pedicellata* A.C.Sm., Bulletin of the Bernice P. Bishop Museum 141: 61, fig. 29. 1936.

##### Type.

Fiji, Ovalau, near Port Kinnaird, Jul. 1860, *B. Seemann 4* (holotype: K[K000691678]).

#### Meiogyne amygdalina

Taxon classificationPlantaeMagnolialesAnnonaceae

(A.Gray) B.Xue & R.M.K.Saunders, Syst. Bot. 39(2): 401. 2014.

4C26FBB6-1B3B-57CB-B48E-7EAC6537680E

##### Basionym.

*Uvaria amydalina* A.Gray, Bot. U.S. Expl. Exped. 1: 31. 1854.

##### Homotypic synonym.

*Polyalthia amygdalina* A.Gray Gillespie, Bernice P. Bishop Mus. Bull. 83: 4. 1931.

##### Heterotypic synonym.

*Desmos leucanthus* A.C.Sm., J. Arnold Arbor. 31 (2): 156. 1950.

##### Type.

Fiji, Ovalau, 1840, *Wilkes Explor. Exped. s.n.* (hololectotype, designated by Smith (1936: 60): GH[GH00039616]; isolectotype: US[US00104128]).

#### Meiogyne habrotricha

Taxon classificationPlantaeMagnolialesAnnonaceae

(A.C.Sm.) B.Xue & R.M.K.Saunders, Syst. Bot. 39(2): 401. 2014.

458A91D1-8210-51E6-A83C-3BF44DE16D7E

##### Basionym.

*Polyalthia habrotricha* A.C.Sm., J. Arnold. Arbor. 31: 157–158. 1950.

##### Type.

Fiji, Viti Levu, Nandronga & Navosa Province, on the northern portion of the Rairaimatuku Plateau, between Nandrau and Rewasau, 11 Aug. 1947, *A.C. Smith 5614* (holotype: A[A00019830]).

#### Meiogyne insularis

Taxon classificationPlantaeMagnolialesAnnonaceae

(A.C.Sm.) D.C.Thomas, B.Xue & R.M.K.Saunders, Syst. Bot. 39(2): 401. 2014.

5A01BE0E-7681-5D3D-AA15-DF0D2FE2D962

##### Basionym.

Desmos insularis A.C.Sm., Sargentia 1: 31–32. 1942.

##### Homotypic synonyms.

*Polyalthia insularis* (A.C.Sm.) A.C.Sm., Allertonia 1: 351. 1978. Meiogyne stenopetala subsp. insularis (A.C.Sm.) Heusden, Blumea 38: 507. 1994.

##### Type.

Fiji, Viti Levu, Mba Province, east of Tavua, near Korovou, 1 Apr. 1941, *O. Degener 14968* (holotype: A[A00019829]; isotypes: BISH[BISH1000666], F, K[K000691250], L[L0037996], MICH, P[P00636931], S, US, WIS).

#### Meiogyne laddiana

Taxon classificationPlantaeMagnolialesAnnonaceae

(A.C.Sm.) B.Xue & R.M.K.Saunders, Syst. Bot. 39(2): 401. 2014.

8E7C3314-CC39-519B-9834-CBB031B18682

##### Basionym.

*Polyalthia laddiana* A.C.Sm., Bernice P. Bishop Mus. Bull. 141: 60–61, fig. 28. 1936.

##### Type.

Fiji, Fulanga, 22 Feb. 1934, *A.C. Smith 1147* (holotype: BISH; isotypes: GH[GH00039621], K[K000691674], NY[NY00026208], P[P00636929], S[S07-13360], US[US00098666], WIS[WIS00000302MAD]).

### Key to *Goniothalamus*, *Huberantha* and *Meiogyne* in Fiji

**Table:** 

1	Flowers with inner petals connivent, forming a mitriform dome over the reproductive organs	*** Goniothalamus ***
–	Flowers with inner petals spreading	**2**
2	Inner petals adaxially grooved at the base; staminal connectives with a tongue-shaped apical prolongation in innermost stamens; 1 to many seeds per monocarp	*** Meiogyne ***
–	Inner petals not grooved; staminal connectives of innermost stamens not expanded; 1 seed per monocarp	*** Huberantha ***

### Key to species of *Huberantha* in Fiji

**Table:** 

1	Leaf blade narrowly lanceolate; monocarps ellipsoid; stipe c. 10–20 mm long	***H. amoena***
–	Leaf blade ovate or broadly lanceolate; monocarps oblong; stipe less than 10 mm long	**2**
2	Leaf base obtuse, petiole 8–12 mm long	***H. capillata***
–	Leaf base rounded or subcordate, petiole 2–6 mm long	**3**
3	Young branches and leaves often persistently yellowish-hirsute	***H. loriformis***
–	Young branches and leaves glabrous	***H. vitiensis***

## Supplementary Material

XML Treatment for Goniothalamus angustifolius

XML Treatment for Huberantha amoena

XML Treatment for Huberantha capillata

XML Treatment for Huberantha loriformis

XML Treatment for Huberantha vitiensis

XML Treatment for Meiogyne amygdalina

XML Treatment for Meiogyne habrotricha

XML Treatment for Meiogyne insularis

XML Treatment for Meiogyne laddiana

## Appendix 1

Voucher information and GenBank accession numbers for samples used in this study (—, missing data; *, newly generated sequences). Voucher data are given for accessions for which DNA sequences were newly obtained, using the following format: species, origin, voucher and Genbank accession numbers for *matK*, *rbcL* and *trnL-F*. For DNA sequences published in previous studies, voucher information is available from GenBank.

***Alphonsea elliptica*** Hook.f. & Thomson, AY518807, —, AY319078; ***Ambavia gerrardii*** (Baill.) Le Thomas, AY220435, —, AY220411(intron), AY220358(spacer); ***Anaxagorea silvatica*** R.E.Fr., AY743477, AY743439, AY743458; ***Annickia chlorantha*** (Oliv.) Setten & Maas, AY841393, AY841594, AY841671; ***Annona glabra*** L., DQ125050, AY841596, AY841673; ***Asimina triloba*** (L.) Dunal, AY743479, AY743441, AY743460; ***Cananga odorata*** (Lam.) Hook.f. & Thomson, AY841394, AY841602, AY841680; ***Cleistopholis glauca*** Pierre ex Engl. & Diels, AY841395, AY841603, AY841681; ***Dasymaschalon macrocalyx*** Finet & Gagnep., EF179277, AY841610, AY841688; ***Dendrokingstonia nervosa*** (Hook.f. & Thomson) Rauschert, KJ418392, KJ418382, KJ418407; ***Desmopsis schippii*** Standl., AY518805, AY319060, AY319174; ***Desmos chinensis*** Lour., JQ768567, JQ762414, JQ762415; ***Fenerivia chapelieri*** (Baill.) R.M.K.Saunders, JF810375, JF810387, JF810399; ***Friesodielsia desmoides*** (Craib) Steenis, JQ768577, JQ768696, JQ768738; ***Goniothalamus angustifolius*** (A.C.Sm.) B.Xue & R.M.K.Saunders, KM818569, KM818797, KM818878; ***Goniothalamus grandiflorus*** (Warb.) Boerl., KM818587, KM818802, KM818851; ***Goniothalamus howii*** Merr. & Chun, KM818590, KM818833, KM818886; ***Goniothalamus majestatis*** P.J.A.Kessler, KM818598, KM818788, KM818903; ***Goniothalamus monospermus*** (A.Gray) R.M.K.Saunders, KM818601, KM818790, —; *Goniothalamus tapis* Miq., DQ125058, AY841622, AY841700; *Goniothalamus wrayi* King, KM818630, KM818803, KM818859; ***Greenwayodendron oliveri*** (Engl.) Verdc., AY743489, AY743451, AY743470; ***Guatteria anomala*** R.E.Fr., AY740913, AY740962, AY741011; ***Huberantha amoena*** (A.C.Sm.) Chaowasku, Fiji, Vanua Levu, A. C. Smith 6423 (A), MW024830*, —, MW024834*; ***Huberantha capillata*** (A.C.Sm.) Chaowasku, Fiji, Vanua Levu, A. C. Smith 4581 (A), MW024831*, —, MW024835*; ***Huberantha cerasoides*** (Roxb.) Chaowasku, AY518854, AY319017, AY319131; ***Huberantha decora*** (Diels) Chaowasku, —, —, JX544869; ***Huberantha henrici*** (Diels) Chaowasku, —, —, JX544870; ***Huberantha jenkinsii*** (Hook.f. & Thomson) Chaowasku, —, —, JX544803; ***Huberantha korinti*** (Dunal) Chaowasku, EU522234, EU522289, EU522179; ***Huberantha loriformis*** (Gillespie) Chaowasku, Fiji, Vanua Levu, J. W. Gillespie 2055 (NY), MW024832*, MW024833*, MW024836﻿*; ***Huberantha nitidissima*** (Dunal) Chaowasku, KF682110, KF682103, KF682105; ***Huberantha pendula*** (Capuron ex G.E.Schatz & Le Thomas) Chaowasku, AY518852, AY319030, AY319144; ***Huberantha perrieri*** (Cavaco & Keraudren) Chaowasku, —, —, JX544871; ***Huberantha stuhlmannii*** (Engl.) Chaowasku, AY518853, —, AY319149; ***Huberantha tanganyikensis*** (Vollesen) Chaowasku, —, —, JX544872; ***Huberantha vitiensis*** (Seem.) Chaowasku, KM924849, KM924919, KM924950; ***Maasia discolor*** (Diels) Mols, P.J.A.Kessler & Rogstad, AY518872, AY319021, AY841584; ***Marsypopetalum crassum*** (R.Parker) BXue & R.M.K.Saunders, HQ286571, HQ286577, HQ286583; ***Meiogyne amicorum*** (A.C.Sm.) B.Xue, D.M.Johnson & R.M.K.Saunders, KF301021, —, KF573503; ***Meiogyne amygdalina*** (A.Gray) B.Xue, D.M.Johnson & R.M.K.Saunders, KF301022, —, KF573497; ***Meiogyne bidwillii*** (Benth.) D.C.Thomas, Chaowasku & R.M.K.Saunders, JQ723764, JQ723851, JQ723904; ***Meiogyne habrotricha*** (A.C.Sm.) B.Xue, D.M.Johnson & R.M.K.Saunders, KF301025, —, KF573498; ***Meiogyne hainanensis*** (Merr.) Bân, JQ723773, JQ723860, JQ723913; ***Meiogyne heteropetala*** (F. Muell.) D.C.Thomas, Chaowasku & R.M.K.Saunders, JQ723766, JQ723853, JQ723906; ***Meiogyne insularis*** (A.C.Sm.) D.C.Thomas, B.Xue & R.M.K.Saunders, KF301028, —, KF573502; ***Meiogyne laddiana*** (A.C.Sm.) B.Xue, D.M.Johnson & R.M.K.Saunders, KF301026, —, KF573499; ***Meiogyne stenopetala*** (F.Muell.) Heusden, JQ723779, JQ723866, JQ723919; ***Meiogyne virgata*** (Blume) Miq., AY518798, AY318982, AY319094; ***Miliusa cuneata*** Craib, AY518844, —, AY319097; ***Miliusa horsfieldii*** (Benn.) Pierre, AY518849, —, AY319098; ***Miliusa indica*** Lesch. ex A.DC., JQ723781, JQ723868, JQ723921; ***Miliusa thorelii*** Finet & Gagnep., AY518846, —, AY319104; ***Miliusa velutina*** (Dunal) Hook.f. & Thomson, AY518847, AY318993, AY319105; ***Mitrephora alba*** Ridl., AY518855, AY318994, AY319106; ***Monanthotaxis whytei*** (Stapf) Verdc., EF179278, AY841635, AY841713; ***Monocarpia euneura*** Miq., AY518865, AY318998, AY319111; ***Monoon lateriflorum*** (Blume) B.Xue & R.M.K. Saunders, JQ723783, JQ723870, JQ723923; ***Neo-uvaria acuminatissima*** (Miq.) Airy–Shaw, AY518793, AY318999, AY319112; ***Orophea enterocarpa*** Maingay ex Hook.f. & Thomson, AY518815, —, AY319119; ***Phaeanthus splendens*** Miq., AY518864, JX544754, AY319126; ***Piptostigma mortehani*** De Wild., AY743492, AY743454, AY743473; ***Platymitra macrocarpa*** Boerl., AY518812, AY319013, AY319127; ***Polyalthia johnsonii*** (F.Muell.) B.Xue & R.M.K.Saunders, JQ723767, JQ723854, JQ723907; ***Polyalthiopsis floribunda*** (Jovet-Ast) Chaowasku, Chaowasku 168, MG264583, MG264580, MG264575; ***Popowia pisocarpa*** (Blume) Endl., AY518862, AY319044, AY319158; ***Pseuduvaria fragrans*** Y.C.F.Su, Chaowasku & R.M.K.Saunders, JQ723784, JQ723871, JQ723924; ***Sageraea lanceolata*** Miq., AY518799, AY319050, AY319164; ***Sapranthus viridiflorus*** G.E.Schatz, AY743493, AY319051, AY319165; ***Stelechocarpus burahol*** (Blume) Hook.f. & Thomson, AY518803, AY319053, AY319167; ***Stenanona costaricensis*** R.E.Fr., AY518801, AY319069, AY319183; ***Tridimeris*** sp., JX544750, JX544753, JX544782; ***Trigynaea lanceipetala*** D.M.Johnson & N.A.Murray, AY743487, AY743449, AY743468; ***Trivalvaria macrophylla*** (Blume) Miq., HQ286576, HQ286582, HQ286588; ***Uvaria lucida*** Benth., AY238966, AY238957, EF179319; ***Wangia saccopetaloides*** (W.T.Wang) X.Guo & R.M.K.Saunders, KF680920, KF680926, KF680930; ***Wuodendron praecox*** (Hook.f. & Thomson) B.Xue, Y.H.Tan & X.L.Hou, MF687367, MF687373, MF687375.
